# Supplementary material for: Factors Affecting BMI Changes in Mothers during the First Year Postpartum
Source: Nutrients. 2023 Mar 11;15(6):1364. doi: 10.3390/nu15061364 (PMC10051214; doi:10.3390/nu15061364)
Supplement: Supplementary file 1 [file nutrients-15-01364-s001.zip › nutrients-2243984-supplementary.pdf]

**Table S1**. Output of the linear mixed-effects model on BMI trajectories during the first year postpartum that includes the three-way interaction of prepregnancy BMI group, infant feeding modality group, and time[2]

| **Characteristics** | **Coefficient (95% CI)** |
|---|---|
| Age | 0.00 (-0.08, 0.08) |
| Income | -0.66 (-1.24, -0.09) |
| GWG | -0.08 (-0.13, -0.03) |
| Prepregnancy BMI group | |
| Overweight | -0.33 (-2.11, 1.44) |
| Obesity | -0.77 (-2.34, 0.80) |
| Infant feeding group | |
| Non-lactating women | -0.32 (-1.59, 0.95) |
| Time | -1.37 (-1.68, -1.05) |
| Time$^2$ | 0.06 (0.04, 0.08) |
| Prepregnancy BMI x time | |
| Overweight | 0.55 (-0.17, 1.26) |
| Obesity | 1.80 (1.17, 2.43) |
| Prepregnancy BMI x time$^2$ | |
| Overweight | -0.03 (-0.08, 0.01) |
| Obesity | -0.08 (-0.12, -0.04) |
| Infant feeding group x time | |
| Non-lactating group | 0.63 (0.16, 1.09) |
| Infant feeding group x time$^2$ | |
| Non-lactating group | -0.05 (-0.07, -0.02) |
| Prepregnancy BMI x Infant feeding group x time | |
| Non-lactating group x Overweight | 1.47 (0.05, 2.4) |
| Non-lactating group x Obesity | -0.03 (-0.82, 0.08) |
| Prepregnancy BMI x Infant feeding group x time$^2$ | |
| Non-lactating group x Overweight | -0.07 (-0.12, -0.01) |
| Non-lactating group x Obesity | 0.02 (-0.03, 0.07) |

*n*= 206/208 because income was missing for two women.
Lactating women and healthy weight women are the reference groups for the model. The interactions did not improve the fit of the model (Bayesian information criterion: 9,877 with vs. 9,845 without the interaction).
Abbreviations: BMI: body mass index; GWG, gestational weight gain
